# Satisfaction and self-confidence among nursing students with simulation learning during COVID-19

**DOI:** 10.1186/s12912-023-01489-1

**Published:** 2023-09-22

**Authors:** Mohammad Alsadi, Islam Oweidat, Huthaifah Khrais, Ahmad Tubaishat, Abdulqadir J. Nashwan

**Affiliations:** 1 Faculty of Nursing, Zarqa University, P. O. Box 132222, Zarqa, 13132 Jordan; 2 Faculty of Nursing, Al al-Bayt University, P. O. Box 130040, Mafraq, 25113 Jordan; 3 Nursing for Education & Practice Development, Hazm Mebaireek General Hospital (HMGH), Hamad Medical Corporation (HMC), University of Calgary in Qatar (UCQ), Doha, Qatar

**Keywords:** Satisfaction, Self-confidence, Simulation, Undergraduate students, Nursing

## Abstract

**Aim:**

This survey aimed to investigate nursing students’ satisfaction and self-confidence in simulation in education during the COVID-19 pandemic. Along with comparing these levels based on selected students’ characteristics.

**Design:**

A cross-sectional survey.

**Methods:**

The survey was conducted at the faculty of nursing of a private university in Jordan. Students’ satisfaction and self-confidence levels in simulation learning were measured using the National League for Nursing (NLN) Student Satisfaction and Self-confidence in Learning Scales.

**Results:**

A total of 138 undergraduate nursing students participated in the survey. Students’ satisfaction levels and self-confidence in simulation learning were lower (just above the scale’s midpoint) than scores reported in similar surveys. The lowest student ratings were reported as “the variety of learning activities that can be done using simulation” and “the self-confidence to develop the needed skills and knowledge to be used in real clinical settings”. The results also indicated that as students’ progress in the bachelor’s degree program, they develop higher levels of self-confidence in simulation-based learning.

**Conclusions:**

Nursing students’ experience of simulation learning was observed to be negatively affected by the COVID-19 pandemic. High-fidelity simulation, in particular, among other simulation modalities, can be more beneficial in similar situations. Education stakeholders are invited to invest in the resources of high-fidelity simulation to maximize its benefits and help in the recovery phase after the pandemic.

## Introduction

With the complexity of healthcare systems and rapidly changing healthcare delivery environments, healthcare professionals need to acquire the knowledge and skills to meet emerging demands [1]. During educational preparation, being a “safe” practitioner, by meeting the minimum competencies at the entry-level to practice settings, was always set as the main goal [2]. Preparation for such outcomes starts early in educational programs by exposing students to different clinical situations with different levels of complexity [3]. Simulation was proposed to be among many solutions to better prepare nursing students, as it offers a controlled setting to practice clinical skills, with its related clinical decision making, in a safe environment [4]. Simulation holds no risk to students or simulated patients if clinical errors are made by practicing students [3].

The concept of simulation was defined by the Agency for Healthcare Research and Quality (AHRQ) at their healthcare simulation dictionary, second edition 2.1 [5] as “A technique that creates a situation or environment to allow persons to experience a representation of a real health care event for the purpose of practice, learning, evaluation, testing, or to gain understanding of systems or human actions (Society for Simulation in Healthcare). However, in healthcare education, i.e., nursing, this definition may give a wide range of what can be named a “simulation” and how it can be conducted. Alinier (2007) described the different simulation modes based on educational needs and the available tools and technology [6]. Alinier then concluded six types of simulation in healthcare education. They are written simulations, three-dimensional models, screen-based simulators, standardized patients, intermediate fidelity patient simulators, and interactive patient simulators. Then, with the advancement of simulation technology and the reported evidence of its supportive role in the different fields of healthcare education, including nursing, more attention was paid to investing in the latest and advanced High Fidelity Simulation (HFS) [7]. HFS is defined as a “technology-based educational approach performed in a realistic and safe environment that uses an interactive patient simulator able to reproduce life-like clinical conditions allowing students to improve their technical and nontechnical skills” [8].

Simulation “design” was described by the National League for Nursing (NLN) Jeffries’ simulation theory as one of the main factors that affect the process and the outcomes of using simulation in nursing education [9]. The simulation design as described by Jeffries and colleagues incorporate the factors from the learner perspective which include having clear objectives and information about the simulation activity, presence of enough support to run the activity, developing the problem-solving abilities, feedback and the level/modality of fidelity (realism) in the simulation activity. During COVID-19 pandemic these factors came in into play as the simulation-based learning was enforced. Optimizing and potentiating all of the factors (dimensions) of the simulated activity may increase the likelihood to achieve better educational outcomes of the simulation activities.

Using simulation in nursing education was found to have many benefits. It includes better nursing students’ self-confidence and satisfaction at the clinical areas [10], better clinical decision-making skills [3], and a higher level of competence in acquiring cognitive knowledge and performing the needed clinical psychomotor skills [11]. However, despite the presented evidence, it was observed by the research team in the current survey that COVID-19 pandemic restrictions lead to observed frustration of nursing students being unable to access the clinical settings, then; the authors noticed that the utilization of clinical settings replacement, i.e. simulation, was not enough to have a good satisfaction and self-confidence in simulation learning compared to pre-COVID-19 era where students showed more excitement, satisfaction and self-confidence in simulation-based learning. This comes in despite the fact that the current students are of generation z (born 1995 and after). The generation is defined to be technology-bound and easy to adopt technology as a method of engaging into learning activities. They are frequently called “iGeneration”, or “Digital natives” [12]. The observations were based on nursing students verbalizing their frustrations related to simulation learning methods during the COVID-19 pandemic. The observation made by the study authors can be related to the contextual enforced situation that simulation-based learning was the “only” available option during the pandemic, compared to the “additional” method of learning at the regular conditions where most of training takes place at real clinical settings. Moreover, it can be due to the fear of students of being incompetent to deal with real-life situations after going through COVID-19 era. Thus, the current survey is being conducted to explain the observation in a scientific methodology.

The current survey aims to explore nursing students’ levels of satisfaction and self-confidence in simulation learning experiences during the COVID-19 pandemic. The objectives were proposed to measure the levels of satisfaction and self-confidence in simulation, to compare the levels based on different nursing students’ demographics, including computer skills as it was reported in literature as a correlate of nurses’ utilization of technology including simulation-based learning [13]. Then, to evaluate the strength and direction of the relationship with satisfaction and self-confidence.

## Methods

### Design and setting

A cross-sectional self-report survey was utilized at the faculty of nursing of one private university in Jordan. For the purpose of this survey, there are two main modalities of simulation that students participated in. One is a mixture of simulation modalities from written simulations (case scenarios) to high-fidelity simulations in the clinical training laboratories and the other modality was remote simulation modules that can be run online and controlled by the course instructor. High fidelity simulation laboratories were well-equipped to cover a wide range of modules that mimics many clinical wards and units. Due to COVID-19 restrictions in prohibiting mass gatherings, classrooms, and the access of nursing students to clinical settings, both simulation modalities were used to conduct the different theoretical and clinical courses in the targeted survey setting in Jordan. The use of the two methods of simulation was distributed in almost a similar manner across the different levels and the different courses to distribute the available resources in all courses and all students. The average use of both methods was at least once daily for most of the courses. All instructors of the courses received the training on using simulation equipment by the company which supplies it. Moreover, the simulation laboratories are the located at the same location of the faculty of nursing clinical laboratories and lecture rooms. Students are familiar with the setting and its equipment even before COVID-19 pandemic which can be considered a psychologically “safe” environment that is familiar to students.

### Sampling

A non-probability convenience sampling design was utilized in the current survey to recruit nursing students. The minimum needed sample size was calculated through the power analysis procedure described by Cohen [14]. Considering an α of 0.05, power of 0.8, medium effect size, and correlation testing as the highest needed statistical procedure, the minimum required sample was 85 nursing students. The number of potential participants was approximately 350 students, who were all invited to participate. The inclusion criteria included that nursing students should at least completed one semester, about 4 months, of learning using simulation during COVID-19 pandemic.

### Instruments

The survey data collection tool included three parts: a brief introductory paragraph that consisted of a description of the survey objectives, purpose, and a consent statement to participate in the survey. Then, the second part consisted of questions about selected characteristics of participants, including age (in years), gender (male, female), academic year (1st, 2nd, 3rd, 4th ), enrolment type (admission from secondary school or Licensed Practical Nurse (LPN) to Registered Nurses (RN) program), the use of simulation in clinical courses (yes/no), the use of simulation in theoretical courses (yes/no), and a Likert-type single-item self-rating scale of computer skills ranging from “1” as a “novice” in using computers and “7” which is “expert” in using computers adopted from Staggers Nursing Computer Experience Questionnaire [15].

The third section consisted of the widely used 13-item Student Satisfaction and Self-Confidence in Learning Scale developed by the National League for Nursing (NLN) in 2006, where 5 items are related to students’ satisfaction in simulation learning scale and 8 items are related to self-confidence in simulation learning scale. The psychometric properties of the scale were tested on comparable populations of nursing students and reported to be valid by running item analysis in subsamples concordant and discordant validity testing by [16]. Moreover, internal consistency was tested, and Cronbach’s alpha was 0.94 for the satisfaction subscale and 0.87 for the self-confidence scale [16]. It was also confirmed by Unver and colleagues (2017) that the internal consistency was tested and reported a Cronbach’s alpha of 0.77–0.85, which is also above the acceptable level of 0.7 [17].

The responses for each item of the scale range between “1 = Strongly disagree” and “5 = Strongly agree”. The mean score of all participants for each of the items was calculated. Additionally, the total mean score for the satisfaction and self-confidence subscales was also calculated with a possible range between 1 and 5. A higher mean score for the subscales indicates higher satisfaction and higher self-confidence with simulation-based education. Despite the fact that the NLN tool is among the most widely used tools to assess novice nurses and nursing students’ satisfaction and self confidence toward simulation-based learning, it is reported in literature that its assessment is limited to the lower levels of students’reaction to simulation-based learning and does not evaluate the ultimate outcome of simulation-based learning on nurses’ psychmotor skills which will affect the provided care to patients [18].

### Data collection procedure

Data were collected online using MS Teams™ forms. The link to reach the form was made open to all nursing students through the university Teams™ groups at the faculty of nursing. It requires the students to use their access credentials to reach the form and complete it. The principal researcher contacts were made available to all students and faculty members if any inquiries were raised. At the same time, students’ responses were made anonymous even to the research team. Reminders were sent to students through the MS Teams™ groups. Upon reaching 138 responses, the form was made unavailable to students to respond to, and a “Thank you” notification was sent though MS Teams™ groups. The responses for each question on the form were made mandatory to submit; thus, no missing data were encountered. Additionally, to maintain ethical and voluntary participation, participants can withdraw from completing the form at any time.

### Ethical considerations

Ethical approval to conduct this survey was granted by the Institutional Review Board (IRB) at the faculty of nursing at Zarqa University under approval number [13/2021]. The survey ensured the voluntary participation of students and the right to withdraw at any time without any consequences. Electronic data were stored in a password-protected computer to maintain the privacy and confidentiality of the participants.

## Results

After completing the data collection, responses were extracted as an MS Excel™ sheet and then entered into SPSS™ version 23.0 for data analysis. Descriptive statistics were conducted using frequencies, percentages, and calculations of scales’ means. Assumptions to run inferential statistics were also conducted and met (i.e. normal distribution of continuous variables, homogeneity of variance, and mutually exclusive groups. Then, a t-test was used to compare the mean scores of the two main dependent variables, satisfaction and self-confidence, when comparing between the two groups within any of the independent variables. ANOVA was used to compare the mean scores whenever the independent variable divides the participants into three groups or more. Finally, Pearson’s product moment correlation was used to test the relationships between satisfaction, self-confidence, and computer skills (Bowers, 2019).

### Sample characteristics

One hundred thirty-eight bachelor’s degree students at the faculty of nursing at one private university in Jordan participated in the survey, out of the 350 students who were invited to participate. The response rate was calculated to be 39.4%. The results show that students’ mean age was 22 years (SD = 3.2), which put them into generation Z (born 1995 and later). More female students participated in the survey than males (n = 81, 58.7%). Students belonged to different academic levels, and second-year students were the largest participants in this survey (n = 51, 37%). Since two tracks are available for nursing students in Jordan to enroll in nursing programs, students in this survey mainly enrolled by the direct admission track after secondary school (78.3%, n = 108). Regarding computer skills, nursing students rated themselves to be at a “good” level (M = 5, SD = 1.3) in computer skills using a 1 to 7 self-rating scale (Table 1).

**Table 1:** Demographical characteristics

Characteristic	N (%)
Age M (SD)	22 (3.2)
Range	19–33
Gender	
Male	57 (41.3)
Female	81 (58.7)
Academic level	
1st Year	18 (13.0)
2nd Year	51 (37.0)
3rd Year	45 (32.6)
4th Year	24 (17.4)
Enrollment Type	
Direct admission	108 (78.3)
LPN to RN	30 (21.7)
Preference of using simulation in clinical teaching	
Yes	129 (93.5)
No	9 (6.5)
Preference of using simulation in theory teaching	
Yes	90 (65.2)
No	48 (34.8)
Computer skills M (SD)	5 (1.3)
Range	1–7

M = Mean, SD = Standard Deviation, N = Count.

Based on the distributions of the reported scores, the results showed that most of the students (93.5%, n = 129) preferred simulation-based learning at the clinical training laboratories. Moreover, students’ preference in using remote simulation modules in learning the needed theoretical knowledge drops to 65% (n = 90) (Table 1).

### Levels of satisfaction and self-confidence

Table 2 shows that the students’ total satisfaction with simulation learning was just above the midpoint of the scale (M = 3.1, SD = 1.3). The students reported the highest rating for “enjoying” how the instructors taught them using the simulation in both methods and reported the lowest rating for the variety of learning materials and activities used through simulation in general for both methods.

**Table 2:** Satisfaction and self-confidence in simulation learning

	Satisfaction Statements	
		**Mean (SD)**
1.	I enjoyed how my instructor taught the simulation.	3.4 (1.2)
2.	The way my instructor(s) taught the simulation was suitable to the way I learn.	3.3 (1.2)
3.	The teaching methods used in this simulation were helpful and effective.	3.1 (1.4)
4.	The teaching materials used in this simulation were motivating and helped me to learn.	3.0 (1.3)
5.	The simulation provided me with a variety of learning materials and activities to promote my learning the different classes’ curricula.	2.8 (1.2)
	**Total Satisfaction with Simulation Learning**	**3.1 (1.3)**
	**Self- Confidence Statements**	
1.	It is the instructor’s responsibility to tell me what I need to learn of the simulation activity content during class time.	3.9 (1.0)
2.	My instructors used helpful resources to teach the simulation.	3.7 (1.1)
3.	It is my responsibility as the student to learn what I need to know from this simulation activity.	3.6 (1.6)
4.	I know how to get help when I do not understand the concepts covered in the simulation.	3.5 (1.1)
5.	I am confident that I am mastering the content of the simulation activity that my instructors presented to me.	3.3 (1.0)
	I know how to use simulation activities to learn critical aspects of these skills.	3.3 (1.2)
6.	I am confident that this simulation covered critical content necessary for the mastery of medical surgical curriculum.	3 (1.4)
7.	I am confident that I am developing the skills and obtaining the required knowledge from this simulation to perform necessary tasks in a clinical setting.	2.9 (1.2)
	**Total Self-Confidence with Simulation Learning**	**3.0 (1.2)**

SD = Standard Deviation

Regarding the students’ total self-confidence in simulation learning, Table 2 demonstrates that it was also above the midpoint of the scale (M = 3.0, SD = 1.2). The highest rating was for the statement that students rely on the instructors to tell them what they need to learn of any simulation activity during class time, while the lowest student rating was for their confidence in developing the needed knowledge and skills to perform the necessary tasks in real clinical settings.

### Students’ characteristics and simulation-based learning

The results showed no statistically significant difference between male and female students regarding satisfaction in simulation (t = 0.27, df = 111.9, p = 0.74) or self-confidence in simulation (t = 0.48 df = 109.5, p = 0.64). Regarding the type of admission, the results demonstrated that there is no significant difference between students who are new admissions to the program and LPN to RN students in their levels of satisfaction with simulation learning (t=-1.5, df = 136, p = 0.13) or their levels of self-confidence in simulation learning (t=-1.59, df = 136, p = 0.12). Similarly, there was no statistically significant difference in the students’ satisfaction with simulation learning among the students from different year level at the 4-year nursing program (F = 2.1, df = 3, p = 0.1). However, there was a statistically significant difference among the students at the different year levels in their levels of self-confidence in simulation learning (F = 9.5, df = 3, p < 0.001). Post hoc analysis shows that the higher the year level, the higher the level of self-confidence is reported, where 4th -year students have the highest self-confidence mean (3.4 (SD = 0.97)) (Table 3).

**Table 3:** Demographics and simulation learning

**Characteristic**	**Satisfaction**			Self-Confidence		
	M(SD)	(t/F)	*p*	M(SD)	(t/F)	*p*
***Gender***		0.27	0.74		0.48	0.64
Male	3.1 (1.07)			3.9 (1.05)		
Female	3.1 (0.96)			3.8 (0.91)		
***Academic level***		2.1	0.10		9.5	< 0.001*
1st Year	2.6 (0.79)			2.9 (1.17)		
2nd Year	3.2 (0.98)			3.9 (0.77)		
3rd Year	3.1 (1.09)			4.0 (1.01)		
4th Year	3.4 (0.97)			4.3 (0.56)		
***Enrollment Type***		-1.56	0.13		-1.59	0.12
New admission	3.0 (0.92)			3.8 (0.90)		
LPN to RN	3.4 (1.25)			4.3 (1.16)		

*significant at *p* < 0.05, M = Mean, SD = Standard Deviation

[Insert Table 3 here]

Despite the significant correlation between computer skills and satisfaction using simulation learning (r = 0.28, p < 0.001); Also, the statistically significant correlation between computer skills and self-confidence in simulation learning (r = 0.21, p < 0.05), both relationships were weak in their strength as shown in Table 4. While; on the other hand, a strong positive correlation was reported between satisfaction and self-confidence in using simulation in learning (r = 0.71, P < 0.001). In other words, as students’ self-confidence in using simulation in learning increases, their satisfaction with using it will increase as well, and vice versa.

**Table 4:** Correlation between satisfaction, Self-confidence, and computer skills

	Satisfaction (r)	Self-confidence (r)
**Computer skills (r)**	0.28 (p = 0.001)	0.21 (0.02)
**Satisfaction (r)**	1	0.71 (p < 0.001)
**Self-confidence (r)**	0.71 (p < 0.001)	1

## Discussion

In the era of the COVID-19 pandemic and the resulting restrictions on higher education, nursing students and educators were obligated not to access clinical settings. Thus, the alternative was to conduct the different theoretical and clinical classes using simulation at the clinical laboratories at the university campus or remotely using online simulation modules. At the current survey, students’ levels of satisfaction and self-confidence in simulation learning were both just above the midpoint of the scales’ possible scores (3.1 and 3.0, out of 5, respectively), which is lower than the results reported by [11, 19–21]. The research team observations about the frequent students’ frustration of using simulation during COVID-19 Pandemic can be one of the reasons to explain the lower scores of students’ satisfaction and self-confidence in the enforced simulation-based learning during COVID-19 pandemic. Also, the lower satisfaction and self-confidence can be attributed to the fear of students of being incompetent to deal with real-life situations after going through COVID-19 era.

At the current survey, the lowest rating was also given to “the limitation in the variety of learning activities that can be done through simulation”. This can be referred to the frustration in the limitation of simulation scenarios compared to real clinical settings regardless of the simulation type being used. While a combined mixture of different modalities of simulation was used for students at the current survey, other studies focused on the importance of integrating and focusing only on high-fidelity simulation in nursing education. High-fidelity simulation (HFS) was proven in the literature to have better educational outcomes in terms of preparing student nurses before reaching clinical settings [11, 19]. However, that is not easily feasible for all nursing students at universities to have it utilized at all clinical courses for such an extended period of time, as it requires an extensive presence of resources, including expensive equipment, enough simulation labs and training over extended periods of time [11, 19]. Moreover, most of the studies that investigated satisfaction and self confidence in using simulation-based learning focused on the self-rating of students and did not conclude the actual competencies required through simulation-based learning especially upon dealing with actual patients at the clinical settings [22]. Another dimension here for the self-report rating of nursing students is Dunning-Kruger Effect (DKE), where novice usually over-rate their confidence and skills and experts tend to underestimate their confidence and skills [23]. In this highlight, despite the expectation that novices may overestimate their confidence and satisfaction, students’ scores of the current study were lower than literature which reflects frustration that can be contextual to COVID-19 pandemic restrictions.

The results of the current survey showed no difference in the levels of satisfaction and self-confidence in simulation between students admitted after secondary school and LPN to RN students. Also, the higher the year level of the students, the higher their self-confidence in simulation learning, which is congruent with the results reached by [21]. This can be explained by the duration of exposure to real clinical settings during the program courses prior to the COVID-19 pandemic. The higher the year level, the higher the duration that the student spent at real clinical settings during the bachelor’s degree program. Then, when high reliance on simulation came in, a possible explanation could be that higher experience students could be more capable of connecting what they learned in simulation to a real-life situation they faced before COVID-19. Previous exposure to clinical settings at the LPN level for LPN to RN students did not make a difference in the levels of satisfaction of self-confidence, which may be related to the different levels of competencies needed between the two levels of practice: LPN and RN.

Another dimension was shown in the current survey results, where nursing students linked their computer skills with the level of beneficial outcomes that can be achieved by learning through simulation. This can be attributed to the generation of students at the current survey (Generation Z). Students perceived that the higher their computer skills were, the better educational outcomes could be achieved. This perception can be explained by the reliance on multiple electronic devices through the simulation training, which could create a level of anxiety that needs to be dealt with. Shearer et al. [24] reported this issue in a systematic review about anxiety in using simulation and discussed the theme of “unknown”, which describes the student experience in dealing with unknown devices and settings that may lead to an increased anxiety level of the student. A thoughtful preparation, orientation to the setting, and clear instructions about the scenario are among the steps to reduce the level of students’ anxiety in using simulation and thus maximize its benefits.

### Conclusion and recommendations

The current survey highlights one dimension of pandemic inflictions on nursing education: the use of simulation technology. Simulation-based learning was presented in literature to be effective and promising ahead of the pandemic. With the enforced use of simulation in nursing education, more frustration can be brought to students’ educational experience, which may negatively affect the satisfaction and self-confidence in learning the needed clinical and theoretical knowledge using simulation technology, especially if it is not the high-fidelity modality. Possible explanations of the resulting modest levels of satisfaction and self-confidence with simulation can be referred to the global frustration of the pandemic and its inflictions on all aspects of life, enforcing the simulation on students; not having the “additional” leisure of simulation. More investigation is suggested to elaborate more on such arguments.

Healthcare researchers are also invited to investigate the effects of the COVID-19 pandemic on simulation learning and training in multiple healthcare disciplines and other industrial, applied, and social disciplines. The current survey results may also open the door to conducting qualitative studies to explore the lived experiences of students and instructors as well as using simulation technology during COVID-19 pandemic. In addition to looking into integrating high fidelity simulation in nursing education, a thoughtful decision whether to make it obligatory for certain specified courses or for all clinical nursing education courses.

### Limitations

Despite the strengths presented in conducting the current survey, limitations were also faced. The survey was conducted using a non-probability convenience sampling design at one university in Jordan; this inflicts caution regarding the generalizability of the results. Moreover, using a one group post-test design may limit the full exposure of nursing students’ lived experience with simulation-based learning during COVID-19. The use of the NLN scale in measuring the studetns’self report of self-confidence and satisfaction has its limitation. It does not actually assess the behavioral outcomes as nursing skills to deal with real patients [18]. Another limitation of the study can be Dunning-Kruger Effect, where novices may overrate their confidence and skills, which is the case of the current study sample of nursing students, compared to experts who tend more to lower their self-report evaluation of their confidence and skills. More objective, longitudinal, experimental or qualitative studies may overcome such a limitation in future studies.

## Data Availability

The authors confirm that the data supporting the findings of this study are available within the article.
